# Inverse association of alcohol consumption patterns with constipation and diarrhea: a population-based cross-sectional study

**DOI:** 10.1186/s12876-025-04464-z

**Published:** 2025-12-18

**Authors:** Haoyan Zhuang, Zhao Guo, Shiguo Li, Qixu Fu

**Affiliations:** 1 Anorectal Department, Weifang Hospital Of Traditional Chinese Medicine, Weifang, China; 2 Anorectal Department, Weifang Hospital Of Traditional Chinese Medicine, Shandong Second Medical University, Weifang, China; 3 First Clinical Medical College, Shandong University of Traditional Chinese Medicine, Jinan, China

**Keywords:** Drinking patterns, Constipation, Diarrhea, Negative correlation, NHANES

## Abstract

**Background:**

Constipation and diarrhea are prevalent gastrointestinal health issues that significantly impact productivity and daily life, often leading to psychological problems and exacerbating public health burdens. Alcohol is a commonly consumed beverage worldwide, and previous studies suggest that it may exert certain effects on the gastrointestinal tract. Investigating the relationship between patterns of alcohol consumption and the presence of gastrointestinal symptoms such as constipation or diarrhea can aid in the formulation of public health strategies and improve management plans for alcohol use and gastrointestinal health.

**Objective:**

To explore the relationship between patterns of alcohol consumption (average daily alcohol consumption and drinking levels) and the presence of gastrointestinal symptoms such as constipation or diarrhea.

**Methods:**

The study utilized data from the National Health and Nutrition Examination Survey (NHANES) database from 2005 to 2010, enrolling a total of 12,959 eligible participants aged 20 years and older. Multivariate logistic regression analysis was employed to investigate the association of drinking patterns with constipation and diarrhea. Restricted cubic spline (RCS) curves were used to confirm the nonlinear relationship between these variables. Finally, subgroup analyses were conducted to identify high-risk groups and populations with specific effects.

**Results:**

In this study, a negative correlation was observed between the average daily alcohol consumption and constipation (OR: 0.94, 95% CI: 0.91-0.97; *P* < 0.001). Similarly, a negative correlation was found between drinking levels and constipation (*P* < 0.001). These results remained stable even after adjusting for all confounding factors. However, the negative correlation between both average daily alcohol consumption and drinking levels with diarrhea was only present in model 1, and the results became unstable after adjusting for all confounding factors.

**Conclusion:**

Our study reveals a negative correlation between drinking patterns and both constipation and diarrhea. However, this association warrants further biological research for validation.

## Introduction

Constipation is a prevalent disorder affecting intestinal health and represents a significant health burden globally. Meta-analyses reveal that the worldwide prevalence of constipation is approximately 14%, with women being twice as likely to be affected as men [1]. Additionally, constipation shows a positive correlation with age and is more commonly observed in populations with lower socioeconomic status [1–3].According to studies utilizing the Rome IV diagnostic questionnaire, the prevalence of chronic constipation is approximately 9%. Of this, around 6% is classified as functional constipation, while 3% is attributed to opioid-induced constipation and constipation-predominant irritable bowel syndrome (IBS-C)[4, 5]. Clinically, patients with constipation exhibit symptoms such as reduced bowel movement frequency, hard or lumpy stools, abdominal pain or bloating, straining during defecation, a sensation of incomplete evacuation, and occasionally the need for digital assistance during bowel movements. According to the Rome IV criteria, a diagnosis of functional constipation can be made when two or more of these symptoms occur in over 25% of defecations within the past six months, with active symptoms present for at least three months [6]. Chronic constipation significantly impacts patients’ quality of life and exacerbates the socioeconomic burden [7, 8].

Diarrhea, as another factor affecting intestinal health, caused approximately 1.17 million deaths globally in 2021, posing a serious threat to human life and safety [9]. Diarrhea is typically defined by increased bowel movement frequency, stool water content, volume, and weight. It often requires differentiation from fecal incontinence [10]. The prevalence of diarrhea varies significantly across different regions. In the United States, the prevalence rate is approximately 26.9%. In contrast, the prevalence of functional diarrhea in China is substantially lower than that in Western countries, yet higher than in other parts of Asia[11, 12].. Diarrhea is commonly caused by infections from certain bacteria and viruses, which can easily lead to public health incidents. It poses a serious threat to regions with poor healthcare conditions, as well as low-income and marginalized populations [13].

Alcohol is a common beverage with addictive potential. According to the World Health Organization statistics on individuals aged 15 and above, the average per capita consumption of pure alcohol in the United States in 2020 was 9.90 L. In 2019, the average per capita consumption of pure alcohol for American males was 15.03 L, while for females it was 2.49 L. Between 2006 and 2016, the proportion of American adults who consumed alcohol was 71.7%. In 2021, the prevalence of alcohol or substance use disorders in the United States was 5.9%[14]. Alcohol undergoes first-pass metabolism in the stomach and liver, is absorbed in the small intestine, and is oxidatively metabolized through pathways such as alcohol dehydrogenase (ADH), the microsomal ethanol oxidizing system (MEOS), and the catalase pathway. It also undergoes non-oxidative metabolism, producing compounds such as ethyl glucuronide (EtG), ethyl sulfate (EtS), fatty acid ethyl esters (FAEEs), and phosphatidylethanol (PEth). In the brain, ethanol oxidation is predominantly mediated by catalase. Finally, through the synergistic action of the gut and liver, the majority of alcohol is eliminated in the intestine [15]. The relationship between drinking patterns and gut health remains unclear. Previous studies have mostly focused on single outcomes. Some research has explored the association between alcohol consumption and the risk of constipation [16], while others have examined post-drinking diarrhea as a background factor in assessing the risk of colorectal cancer [17]. Additionally, some studies have considered alcohol consumption as a mediating variable when investigating the relationship between gastrointestinal health and other exposure factors [18]. However, there is a scarcity of research that examines, within the same population framework and using a standardized methodological approach, the relationship between alcohol consumption and two distinct gastrointestinal dysfunctions: constipation and diarrhea. The gut microbiota plays a central role in intestinal health, and alcohol along with its metabolites can significantly impact the ecology of the gut microbiome. Research has found that long-term alcohol consumers have an abundance of *Proteobacteria* of the class *Gammaproteobacteria* and *Firmicutes* of the class *Bacilli*, while there is a reduction in *Firmicutes* of the class *Clostridia* [19]. The aforementioned changes in the microbiota can lead to impairment of the gastrointestinal barrier and disruption of digestive functions, resulting in the occurrence of constipation or diarrhea. Our study utilized data from the National Health and Nutrition Examination Survey (NHANES) database, which includes a nationally representative sample with comprehensive participant information across various ethnicities and age groups. Therefore, based on the NHANES database, we investigated the relationship between drinking patterns and gut health and explored the potential underlying mechanisms.

## Methods

### Study population

**Fig. 1:**
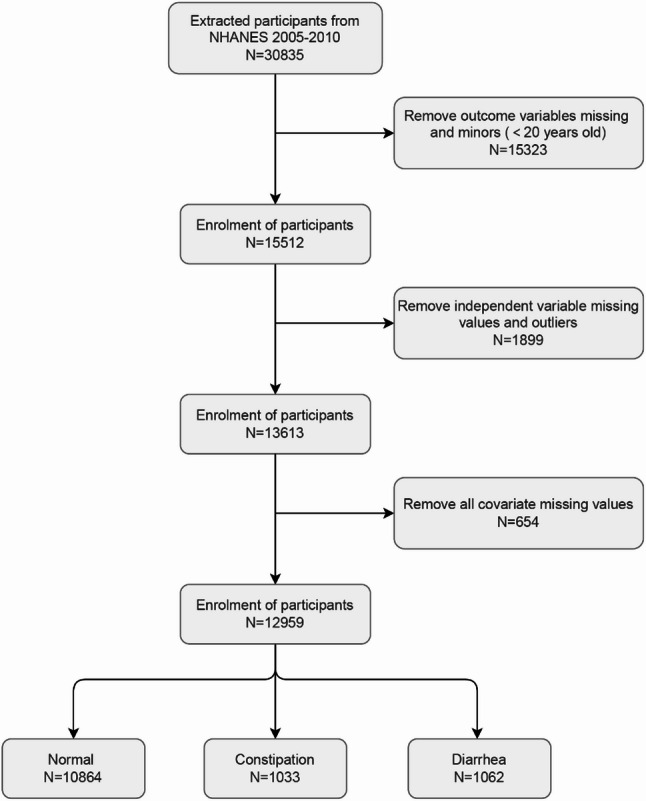
Flowchart of participant selection process

NHANES is conducted by the National Center for Health Statistics (NCHS), a division of the Centers for Disease Control and Prevention (CDC). It provides a standardized examination environment through Mobile Examination Centers (MEC) across the nation. Utilizing a multi-stage, stratified, probability sampling design, NHANES assesses the nutritional and health status of the U.S. population. It includes data from physical examinations, laboratory tests, and personal interviews, as well as data that cannot be reported by individuals or their healthcare providers [20]. The study included data from three NHANES cycles: 2005–2006, 2007–2008, and 2009–2010, comprising a total of 30,835 participants. We excluded 15,323 participants who were under 20 years of age or had missing data from the gastrointestinal health questionnaire. Additionally, we excluded 1,899 participants due to missing or physiologically implausible data in the alcohol consumption questionnaire (e.g., the average number of drinks reported per day was 83), and 654 participants with missing covariate data. Ultimately, the study included 12,959 participants. The detailed selection process is illustrated in Fig. 1.

### Outcomes and exposure factors

The analysis utilized the gastrointestinal health survey from the NHANES database, specifically examining the outcomes based on the Bristol Stool Form Scale (BHQ060). Participants were categorized into distinct groups based on their stool type: Type 1 (separate hard lumps, resembling nuts) and Type 2 (sausage-like, but lumpy) were classified as individuals with constipation. Types 6 (fluffy pieces with ragged edges, a mushy stool) and 7 (watery, with no solid pieces) were designated as those with diarrhea. Types 3–5, which reflect a more typical stool consistency, were considered as indicating normal gastrointestinal health [21].

Alcohol consumption patterns were defined in two components using data from the Alcohol Use Questionnaire (ALQ), wherein “one drink” was standardized as 12 ounces of beer, 5 ounces of wine, or 1.5 ounces of distilled spirits. In the NHANES protocol, “one drink” is standardized and quantified. Therefore, the measure “drinks per day” represents the average daily consumption in these standard units. The first part is the degree of drinking: never drinkers (never had 12 alcoholic beverages in their lifetime or in the past year), light drinkers (women ≤ 1 drink/d, men ≤ 2 drinks/d, or binge drinking 1 day per month [women ≥ 4 drinks/time, men ≥ 5 drinks/time] in the past year), moderate drinkers (women ≤ 2 drinks/d, men ≤ 3 drinks/d, or binge drinking 2 to 5 days per month), heavy drinkers (women ≥ 3 drinks/d, men ≥ 4 drinks/d, or binge drinking ≥ 5 days/month)[22, 23].The second component assessed the average daily alcohol consumption, calculated based on responses to item ALQ130, which queried participants regarding their average number of alcoholic drinks consumed per day over the preceding 12 months. Although this measure does not capture more detailed aspects of drinking patterns, such as drinking frequency, distribution of alcohol intake across different occasions, or whether alcohol is consumed with meals, the average daily intake remains a widely used and standardized metric in epidemiological research for assessing overall alcohol exposure.

### Covariates

In this study, we incorporated a selection of covariates to enhance the robustness of the associations between the outcomes and the exposure factors. These covariates included sex, age, race, educational attainment, marital status, the poverty-to-income ratio (PIR), Body Mass Index (BMI), smoking status, and the presence of various comorbidities, such as diabetes, hypertension, hyperlipidemia, heart failure, coronary artery disease, stroke, cancer, depression (as assessed by the PHQ-9), and sleep disorders.

In the demographic data, age was categorized into three groups: 20–40 years, 41–60 years, and >60 years. Racial background was classified into five categories: Mexican American, Other Hispanic, Non-Hispanic White, Non-Hispanic Black, and Other Race. Educational attainment was divided into three levels: Less than high school, High school or equivalent, and College or higher. The Poverty-to-Income Ratio (PIR) was derived from the Health and Human Services (HHS) poverty guidelines and stratified into three groups: <1.30 (low-income households), 1.30–3.50 (moderate-income households), and >3.50 (high-income households) [24].

In the lifestyle and medical health questionnaire, individuals were classified based on specific criteria: those who answered “yes” to having smoked at least 100 cigarettes in their lifetime were defined as smokers. Participants who responded “yes” to having been informed by a doctor that they have diabetes were defined as having diabetes. Those who answered “yes” to having been told they have high blood pressure were classified as having hypertension. Respondents who answered “yes” to a doctor’s diagnosis of high cholesterol were considered to have hyperlipidemia. Individuals who confirmed having been told by a doctor that they have a sleep disorder were categorized as having a sleep disorder. Lastly, participants who answered “yes” to having been diagnosed with congestive heart failure, coronary artery disease, stroke, or cancer by a physician were classified as having these respective chronic diseases. These definitions provide a standardized framework for categorizing participants based on their medical and lifestyle histories, which is essential for accurately analyzing the associations between these factors and health outcomes.

In the depression assessment survey, the PHQ-9 scale was employed for evaluation, with respondents categorized into four groups based on their scores: 0–9 (no depression), 10–14 (mild depression), 15–19 (moderate depression), and ≥ 20 (severe depression) [25].

BMI was classified according to the World Health Organization (WHO) criteria, expressed in kg/m², and categorized into four groups: <18.5 (underweight), 18.5–24.9 (normal weight), 25.0–29.9 (overweight), and ≥ 30 (obese) [26].

### Statistical analysis

This study utilized R software (version 4.4.3, http://www.r-project.org) for statistical analysis, with a significance level set at *P* < 0.05. Categorical variables were described as percentages and analyzed using either the Chi-square test or Fisher’s exact test. Continuous variables are described using mean ± standard deviation or median and interquartile range, and analyzed using either the T-test or the Mann-Whitney U-test. To investigate the relationships between outcomes and exposures, we employed multivariate logistic regression, treating daily alcohol consumption (drinks/day) as a continuous variable and drinking level as a categorical variable, separately examining their associations with gut health (constipation/diarrhea). Confounding variables were selected based on existing knowledge in the literature and clinical relevance of drinking behavior and gastrointestinal function. In Model 1, no confounders were adjusted for; Model 2 adjusted for age, gender, education, marital status, race, PIR, and BMI; Model 3 was a fully adjusted model, accounting for the following confounding factors: age, gender, education, marital status, race, PIR, BMI, smoking, hypertension, diabetes, hyperlipidemia, heart failure, coronary heart disease, stroke, cancer, sleep disorders, and depression. We developed two separate models to analyze daily alcohol consumption and drinking levels. The two exposure factors were not included in the same model to avoid multicollinearity. To explore the potential nonlinear relationship between average daily alcohol consumption and the risk of constipation and diarrhea, we used restricted cubic splines (RCS) with 5 percentile knots. A trend test was used to explore the linear relationship between drinking level and gut health. The *P*-value for linear trend was calculated by treating the ordinal drinking-level variable as a continuous term in the regression models. In subgroup analyses, we examined potential interactions to assess whether the association between drinking patterns and gut health varied significantly across subgroups defined by age, gender, education, marital status, race, PIR, BMI, smoking, hypertension, diabetes, hyperlipidemia, heart failure, coronary heart disease, stroke, cancer, sleep disorders, and depression.

## Results

### Baseline characteristics

A total of 12,959 eligible participants were included in this study. Among participants with constipation and diarrhea, females outnumbered males in both groups. The median age of participants with constipation was 43.00 years, which was slightly lower than that of the normal participants. In contrast, the median age of participants with diarrhea was 49.00 years, which was higher than that of the normal participants. In both groups, the highest proportion of participants identified as Non-Hispanic White. Regarding educational attainment, the proportion of participants with a college degree or higher was the highest in both constipation and diarrhea groups, at 49.52% and 49.29%, respectively. In terms of the PIR, the median score for the constipation group was 2.66, and for the diarrhea group, it was 2.73, both lower than those of the normal participants. However, subgroup analysis revealed that the proportion of participants from middle-to-high-income families was highest in both groups. Regarding depression status, both the constipation and diarrhea groups had a median score of 2.00 on the PHQ-9. Detailed data are presented in Table 1.

**Table 1:** Baseline characteristics of drinking patterns among adults in the united States and gut health

Characteristic	Overall	Normal	Constipation	*p*	Overall	Normal	Diarrhea	*p*
Weighted sample size	457,055,233	419,070,789	37,984,444		452,942,257	419,070,789	33,871,467	
Gender, *n* (%)				< 0.001				0.017
Male	5,782 (48.40%)	5,463 (50.17%)	319 (28.87%)		5,905 (49.65%)	5,463 (50.17%)	442 (43.14%)	
Female	6,115 (51.60%)	5,401 (49.83%)	714 (71.13%)		6,021 (50.35%)	5,401 (49.83%)	620 (56.86%)	
Age(years), Median(IQR)	45.00 (32.00, 57.00)	45.00 (32.00, 57.00)	43.00 (29.00, 57.00)	0.010	45.00 (32.00, 57.00)	45.00 (32.00, 57.00)	49.00 (37.00, 61.00)	< 0.001
Age, *n* (%)				0.149				< 0.001
[20,40)	4,306 (39.77%)	3,898 (39.48%)	408 (42.90%)		4,154 (38.72%)	3,898 (39.48%)	256 (29.23%)	
[40,60)	3,923 (38.90%)	3,590 (39.17%)	333 (35.83%)		3,949 (39.45%)	3,590 (39.17%)	359 (42.83%)	
≥ 60	3,668 (21.34%)	3,376 (21.34%)	292 (21.26%)		3,823 (21.84%)	3,376 (21.34%)	447 (27.94%)	
Race, *n* (%)				0.042				0.037
Mexican American	1,964 (7.69%)	1,816 (7.74%)	148 (7.14%)		2,032 (7.92%)	1,816 (7.74%)	216 (10.15%)	
Other Hispanic	1,261 (4.68%)	1,110 (4.51%)	151 (6.60%)		1,245 (4.60%)	1,110 (4.51%)	135 (5.83%)	
Non-Hispanic White	5,795 (70.57%)	5,344 (71.06%)	451 (65.13%)		5,785 (70.69%)	5,344 (71.06%)	441 (66.07%)	
Non-Hispanic Black	2,376 (11.24%)	2,132 (10.88%)	244 (15.20%)		2,364 (11.02%)	2,132 (10.88%)	232 (12.74%)	
Other Race	501 (5.83%)	462 (5.82%)	39 (5.93%)		500 (5.77%)	462 (5.82%)	38 (5.21%)	
Education, *n* (%)				< 0.001				< 0.001
Less than high school	2,938 (16.23%)	2,629 (15.86%)	309 (20.29%)		3,046 (16.58%)	2,629 (15.86%)	417 (25.45%)	
High school or equivalent	2,915 (24.02%)	2,612 (23.46%)	303 (30.19%)		2,865 (23.59%)	2,612 (23.46%)	253 (25.26%)	
College or above	6,044 (59.76%)	5,623 (60.68%)	421 (49.52%)		6,015 (59.83%)	5,623 (60.68%)	392 (49.29%)	
Marital status, *n* (%)				0.046				0.010
Married	6,213 (56.49%)	5,727 (56.80%)	486 (53.00%)		6,276 (56.80%)	5,727 (56.80%)	549 (56.72%)	
Widowed	891 (4.94%)	789 (4.66%)	102 (8.05%)		905 (4.84%)	789 (4.66%)	116 (6.99%)	
Divorced	1,312 (9.99%)	1,198 (10.00%)	114 (9.86%)		1,333 (10.09%)	1,198 (10.00%)	135 (11.16%)	
Separated	382 (2.26%)	350 (2.30%)	32 (1.81%)		395 (2.40%)	350 (2.30%)	45 (3.68%)	
Never married	2,177 (18.85%)	1,952 (18.68%)	225 (20.69%)		2,091 (18.31%)	1,952 (18.68%)	139 (13.76%)	
Living with partner	922 (7.47%)	848 (7.55%)	74 (6.59%)		926 (7.56%)	848 (7.55%)	78 (7.70%)	
PIR, Median(IQR)	3.17 (1.64, 5.00)	3.23 (1.67, 5.00)	2.66 (1.31, 4.72)	< 0.001	3.17 (1.65, 5.00)	3.23 (1.67, 5.00)	2.73 (1.31, 4.80)	0.004
PIR, *n* (%)				< 0.001				0.006
< 1.30	3,184 (18.34%)	2,825 (17.74%)	359 (24.92%)		3,203 (18.28%)	2,825 (17.74%)	378 (24.86%)	
[1.30,3.49]	4,833 (36.34%)	4,416 (36.21%)	417 (37.74%)		4,826 (36.17%)	4,416 (36.21%)	410 (35.55%)	
≥ 3.50	3,880 (45.32%)	3,623 (46.04%)	257 (37.35%)		3,897 (45.56%)	3,623 (46.04%)	274 (39.59%)	
BMI(kg/m^2^), Median(IQR)	26.70 (23.60, 30.80)	26.90 (23.70, 31.00)	25.80 (22.60, 29.30)	< 0.001	27.00 (23.70, 31.10)	26.90 (23.70, 31.00)	27.50 (23.90, 32.90)	0.089
BMI, *n* (%)				0.014				0.062
< 18.5	147 (1.24%)	124 (1.16%)	23 (2.07%)		147 (1.21%)	124 (1.16%)	23 (1.80%)	
[18.5,24.9]	3,679 (33.51%)	3,286 (32.94%)	393 (39.83%)		3,557 (32.76%)	3,286 (32.94%)	271 (30.49%)	
[25.0,29.9]	4,251 (35.54%)	3,898 (35.47%)	353 (36.22%)		4,231 (35.09%)	3,898 (35.47%)	333 (30.29%)	
≥ 30	3,820 (29.71%)	3,556 (30.42%)	264 (21.88%)		3,991 (30.94%)	3,556 (30.42%)	435 (37.42%)	
Smoking, *n* (%)				0.009				0.002
Yes	5,320 (43.99%)	4,936 (44.52%)	384 (38.09%)		5,469 (45.10%)	4,936 (44.52%)	533 (52.29%)	
No	6,577 (56.01%)	5,928 (55.48%)	649 (61.91%)		6,457 (54.90%)	5,928 (55.48%)	529 (47.71%)	
Diabetes, *n* (%)				0.415				< 0.001
Yes	1,185 (6.85%)	1,076 (6.75%)	109 (7.98%)		1,265 (7.25%)	1,076 (6.75%)	189 (13.46%)	
No	10,514 (91.51%)	9,607 (91.57%)	907 (90.80%)		10,455 (91.09%)	9,607 (91.57%)	848 (85.15%)	
Borderline	198 (1.64%)	181 (1.68%)	17 (1.22%)		206 (1.66%)	181 (1.68%)	25 (1.39%)	
Hypertension, *n* (%)				0.181				0.002
Yes	3,949 (28.66%)	3,647 (28.94%)	302 (25.64%)		4,135 (29.68%)	3,647 (28.94%)	488 (38.93%)	
No	7,948 (71.34%)	7,217 (71.06%)	731 (74.36%)		7,791 (70.32%)	7,217 (71.06%)	574 (61.07%)	
Hyperlipidemia, *n* (%)				0.819				0.832
Yes	5,134 (41.58%)	4,707 (41.63%)	427 (41.06%)		5,199 (41.66%)	4,707 (41.63%)	492 (42.03%)	
No	6,763 (58.42%)	6,157 (58.37%)	606 (58.94%)		6,727 (58.34%)	6,157 (58.37%)	570 (57.97%)	
Heart failure, *n* (%)				0.577				0.184
Yes	296 (1.67%)	267 (1.65%)	29 (1.92%)		303 (1.70%)	267 (1.65%)	36 (2.34%)	
No	11,601 (98.33%)	10,597 (98.35%)	1,004 (98.08%)		11,623 (98.30%)	10,597 (98.35%)	1,026 (97.66%)	
Coronary heart disease, *n* (%)				0.100				0.937
Yes	403 (2.68%)	377 (2.77%)	26 (1.70%)		420 (2.78%)	377 (2.77%)	43 (2.84%)	
No	11,494 (97.32%)	10,487 (97.23%)	1,007 (98.30%)		11,506 (97.22%)	10,487 (97.23%)	1,019 (97.16%)	
Stroke, *n* (%)				0.123				0.006
Yes	356 (2.15%)	311 (2.06%)	45 (3.14%)		359 (2.22%)	311 (2.06%)	48 (4.20%)	
No	11,541 (97.85%)	10,553 (97.94%)	988 (96.86%)		11,567 (97.78%)	10,553 (97.94%)	1,014 (95.80%)	
Cancer, *n* (%)				0.340				0.239
Yes	1,120 (8.98%)	1,022 (9.08%)	98 (7.82%)		1,143 (9.24%)	1,022 (9.08%)	121 (11.15%)	
No	10,777 (91.02%)	9,842 (90.92%)	935 (92.18%)		10,783 (90.76%)	9,842 (90.92%)	941 (88.85%)	
Sleep disorders, *n* (%)				0.200				0.016
Yes	838 (6.96%)	776 (7.09%)	62 (5.46%)		897 (7.40%)	776 (7.09%)	121 (11.18%)	
No	11,059 (93.04%)	10,088 (92.91%)	971 (94.54%)		11,029 (92.60%)	10,088 (92.91%)	941 (88.82%)	
PHQ-9, Median(IQR)	2.00 (0.00, 4.00)	2.00 (0.00, 4.00)	2.00 (0.00, 6.00)	< 0.001	2.00 (0.00, 4.00)	2.00 (0.00, 4.00)	2.00 (0.00, 6.00)	< 0.001
PHQ-9, *n* (%)				< 0.001				< 0.001
≤ 9	10,924 (93.13%)	10,044 (93.72%)	880 (86.66%)		10,929 (93.12%)	10,044 (93.72%)	885 (85.75%)	
[10,14]	634 (4.51%)	544 (4.16%)	90 (8.41%)		644 (4.46%)	544 (4.16%)	100 (8.26%)	
[15,19]	257 (1.84%)	205 (1.61%)	52 (4.32%)		256 (1.78%)	205 (1.61%)	51 (3.84%)	
≥ 20	82 (0.52%)	71 (0.51%)	11 (0.61%)		97 (0.64%)	71 (0.51%)	26 (2.15%)	
Daily alcohol consumption, Median(IQR)	2.00 (1.00, 3.00)	2.00 (1.00, 3.00)	1.00 (0.00, 2.00)	< 0.001	2.00 (1.00, 3.00)	2.00 (1.00, 3.00)	2.00 (0.00, 3.00)	0.017
Drinking level, *n* (%)				0.001				0.003
No Alcohol	2,818 (19.16%)	2,472 (18.44%)	346 (27.12%)		2,812 (19.07%)	2,472 (18.44%)	340 (26.96%)	
Light	4,279 (39.19%)	3,934 (39.31%)	345 (37.89%)		4,254 (38.77%)	3,934 (39.31%)	320 (32.06%)	
Moderate	1,961 (17.61%)	1,824 (17.95%)	137 (13.93%)		1,982 (17.94%)	1,824 (17.95%)	158 (17.87%)	
Heavy	2,839 (24.04%)	2,634 (24.31%)	205 (21.05%)		2,878 (24.22%)	2,634 (24.31%)	244 (23.11%)	

### The relationship between alcohol consumption patterns and constipation

**Fig. 2:**
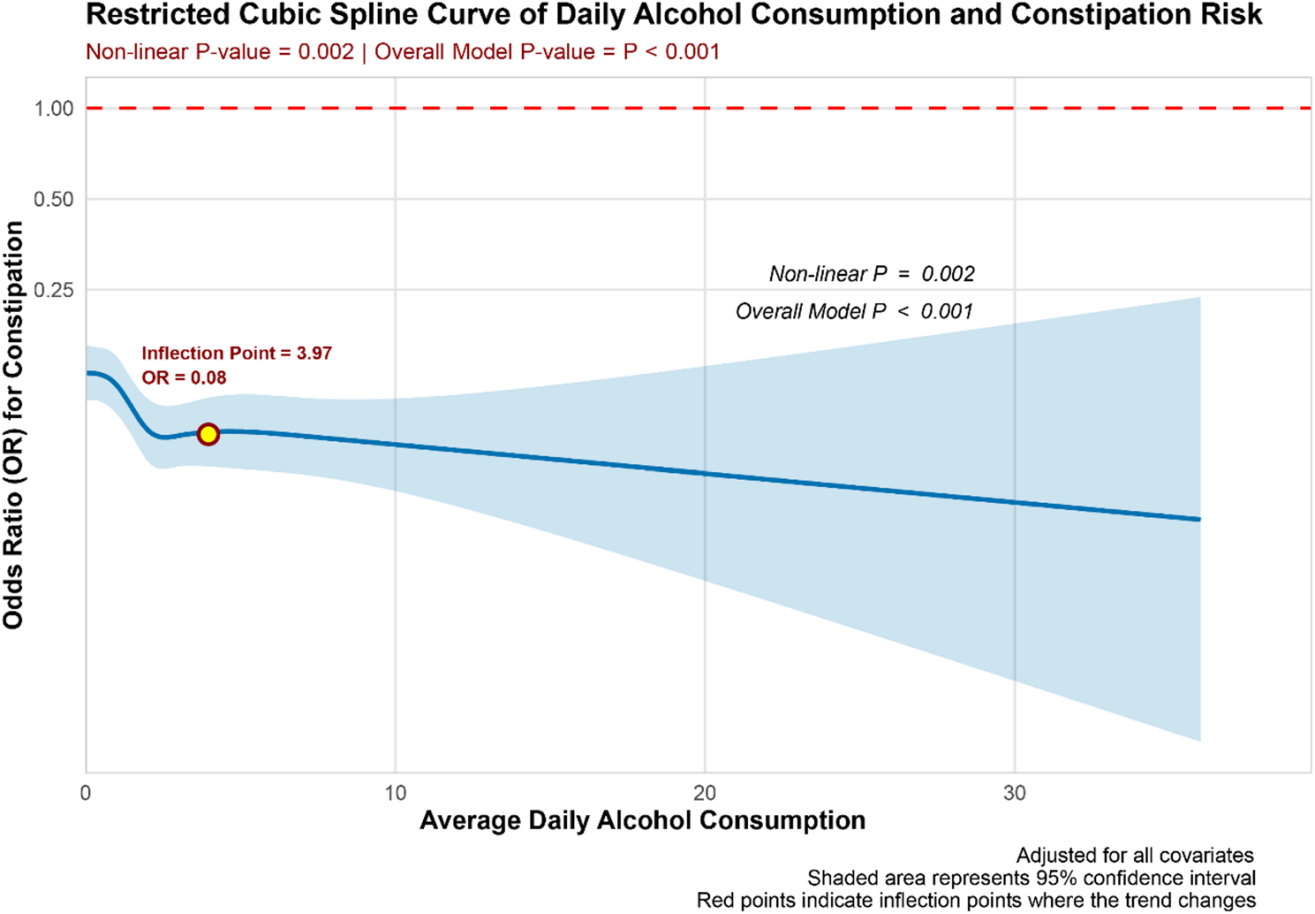
The RCS curve demonstrates the non-linear relationship between average daily alcohol consumption and constipation following multivariate adjustment. The model was adjusted for factors including age, gender, education, marital status, race, PIR, BMI, smoking, hypertension, diabetes, hyperlipidemia, heart failure, coronary heart disease, stroke, cancer, sleep disorders, and depression

Table 2 presents the results of a multivariate logistic regression analysis examining the relationship between alcohol consumption patterns and constipation. In Model 1, which did not adjust for confounding factors, the average daily alcohol consumption was treated as a continuous variable. The results revealed a significant negative correlation between average daily alcohol consumption and constipation (OR: 0.91, 95% CI: 0.88–0.94; *P* < 0.001). This negative association remained statistically significant in both Model 2 (OR: 0.93, 95% CI: 0.90–0.96; *P* < 0.001) and Model 3 (OR: 0.94, 95% CI: 0.91–0.97; *P* < 0.001).In the analysis of drinking levels, light drinking was significantly negatively correlated with constipation in Model 1 (OR: 0.63, 95% CI: 0.54–0.73; *P* < 0.001), and although a negative correlation remained in Models 2 and 3, it did not reach statistical significance. Moderate and heavy drinking were significantly negatively correlated with constipation across all three regression models, with trend tests for all models showing significance (*P* < 0.001). This suggests a trend in which the odds of suffering from constipation decreased with increasing alcohol consumption. Figure 2, after adjusting for all confounding factors, demonstrates through RCS that a non-linear relationship exists between average daily alcohol consumption and constipation (*P* for non-linear = 0.002, *P* for overall model < 0.001).

**Table 2:** Relationship between daily alcohol consumption and drinking levels and constipation

	Model1		Model2		Model3	
	OR(95%CI)	*P*-value	OR(95%CI)	*P*-value	OR(95%CI)	*P*-value
Daily alcohol consumption	0.91(0.88, 0.94)	< 0.001	0.93(0.90, 0.96)	< 0.001	0.94(0.91, 0.97)	< 0.001
Drinking level						
No Alcohol	Reference		Reference		Reference	
Light	0.63(0.54, 0.73)	< 0.001	0.87(0.73, 1.03)	0.107	0.90(0.76, 1.07)	0.225
Moderate	0.54(0.44, 0.66)	< 0.001	0.57(0.46, 0.70)	< 0.001	0.59(0.47, 0.73)	< 0.001
Heavy	0.56(0.46, 0.67)	< 0.001	0.60(0.49, 0.74)	< 0.001	0.64(0.52, 0.79)	< 0.001
*P* for trend		< 0.001		< 0.001		< 0.001

*Abbreviations:* *CI* confidence Interval, *OR *odds ratio

Model1: No covariates adjusted

Model2: Adjusted for Age, Gender, Education, Marital status, Race, PIR, BMI

Model3: Adjusted for Age, Gender, Education, Marital status, Race, PIR, BMI, Smoking, Hypertension, Diabetes, Hyperlipidemia, Heart failure, Coronary heart disease, Stroke, Cancer, Sleep disorders, Depression

### Relationship between drinking patterns and diarrhea

**Fig. 3:**
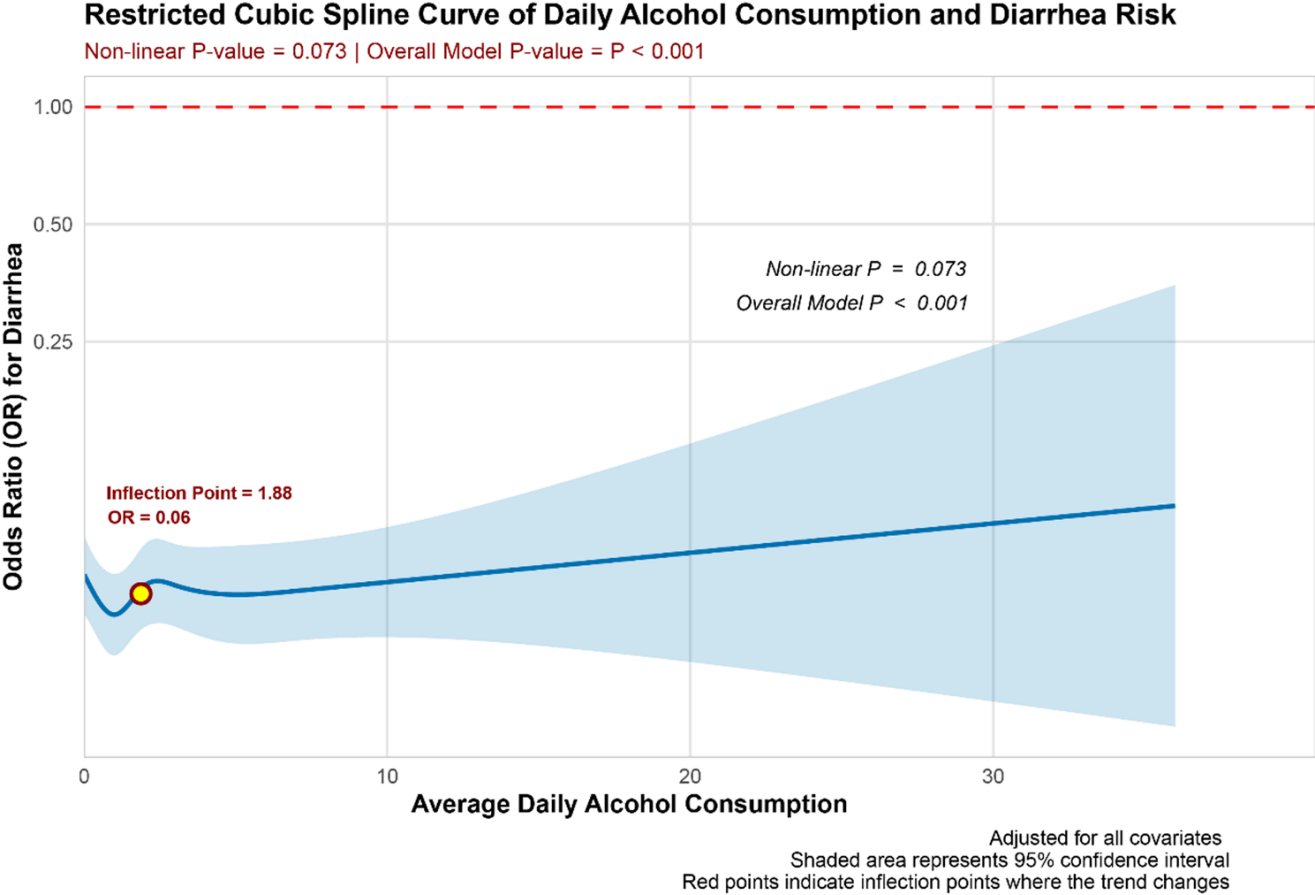
The RCS curve demonstrates the non-linear relationship between average daily alcohol consumption and diarrhea following multivariate adjustment. The model was adjusted for factors including age, gender, education, marital status, race, PIR, BMI, smoking, hypertension, diabetes, hyperlipidemia, heart failure, coronary heart disease, stroke, cancer, sleep disorders, and depression

Table 3 presents the results of a multivariate logistic regression analysis exploring the association between drinking patterns and diarrhea. In Model 1, which did not adjust for confounding factors, daily alcohol consumption was treated as a continuous variable, and a significant negative correlation was observed between average daily alcohol consumption and the incidence of diarrhea (OR: 0.97, 95% CI: 0.94–0.99; *P* = 0.016). However, this relationship was not observed in Model 2 or Model 3. In the analysis of drinking levels, Model 1 revealed a significant negative association between light drinking (OR: 0.59, 95% CI: 0.50–0.69; *P* < 0.001), moderate drinking (OR: 0.63, 95% CI: 0.52–0.77; *P* < 0.001), and heavy drinking (OR: 0.67, 95% CI: 0.57–0.80; *P* < 0.001) and diarrhea. In Model 3, this association remained significant for light drinking, but no significant correlation was found for moderate or heavy drinkers in Models 2 and 3. The trend test in Model 1 was statistically significant (*P* < 0.001), indicating a potential trend towards decreasing diarrhea risk with increasing alcohol consumption. Figure 3, after adjusting for all confounders, shows that the RCS curve did not reveal a significant non-linear relationship between average daily alcohol consumption and diarrhea (*P* for non-linear = 0.073, *P* for overall model < 0.001).

**Table 3:** Relationship between daily alcohol consumption and drinking levels and diarrhea

	Model1		Model2		Model3	
	OR(95%CI)	*P*-value	OR(95%CI)	*P*-value	OR(95%CI)	*P*-value
Daily alcohol consumption	0.97(0.94, 0.99)	0.016	1.01(0.98, 1.04)	0.433	1.00 (0.98, 1.03)	0.786
Drinking level						
No Alcohol	Reference		Reference		Reference	
Light	0.59(0.50, 0.69)	< 0.001	0.85(0.71, 1.01)	0.066	0.82(0.69, 0.98)	0.029
Moderate	0.63(0.52, 0.77)	< 0.001	0.93(0.75, 1.15)	0.510	0.88(0.71, 1.09)	0.251
Heavy	0.67(0.57, 0.80)	< 0.001	1.03(0.84, 1.25)	0.789	0.95(0.77, 1.16)	0.610
*P* for trend		< 0.001		0.651		0.784

*Abbreviations:* *CI* confidence Interval, *OR* odds ratio

Model1: No covariates adjusted

Model2: Adjusted for Age, Gender, Education, Marital status, Race, PIR, BMI

Model3: Adjusted for Age, Gender, Education, Marital status, Race, PIR, BMI, Smoking, Hypertension, Diabetes, Hyperlipidemia, Heart failure, Coronary heart disease, Stroke, Cancer, Sleep disorders, Depression

### Subgroup analysis

#### Subgroup analysis for constipation

**Fig. 4:**
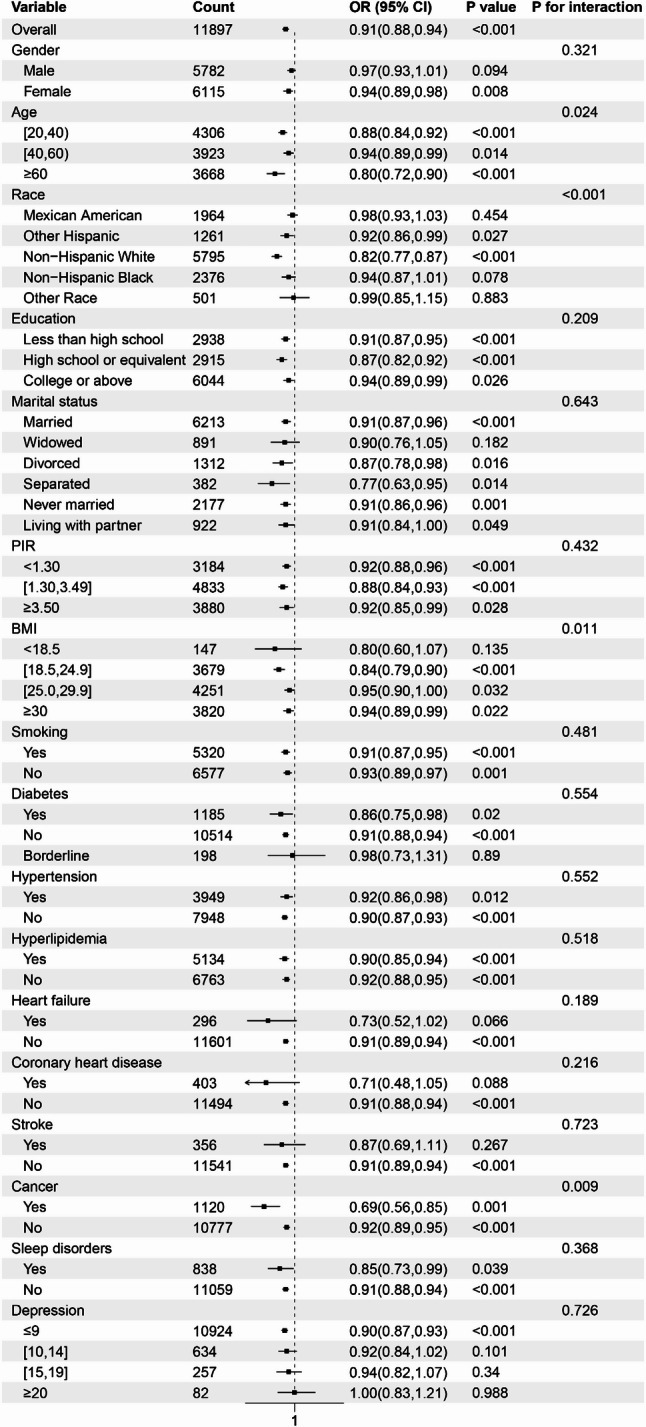
Subgroup analysis illustrating the relationship between drinking patterns and the risk of constipation. The model was adjusted for variables including age, gender, education, marital status, race, PIR, BMI, smoking, hypertension, diabetes, hyperlipidemia, heart failure, coronary heart disease, stroke, cancer, sleep disorders, and depression

Stratified analysis and interaction tests were performed based on factors including age, gender, education, marital status, race, PIR, BMI, smoking, hypertension, diabetes, hyperlipidemia, heart failure, coronary heart disease, stroke, cancer, sleep disorders, and depression. The subgroup analysis revealed that the risk of constipation was lower among individuals in the following categories: youth and elderly (ages 20–40 and ≥ 60), females, non-Hispanic Whites, those with lower educational attainment (college or below), married individuals, middle-to-low-income households (PIR ≤ 3.49), individuals with a BMI between 18.5 and 24.9 kg/m², non-smokers, and those without diabetes, hypertension, heart failure, coronary heart disease, stroke, cancer, sleep disorders, or depression. Notably, the interaction tests for age, race, BMI, and cancer were significant (*P* < 0.05), suggesting that the relationship between drinking patterns and constipation may be influenced by factors such as age, race, BMI, and cancer status. Detailed data can be found in Fig. 4.

#### Subgroup analysis of diarrhea

**Fig. 5:**
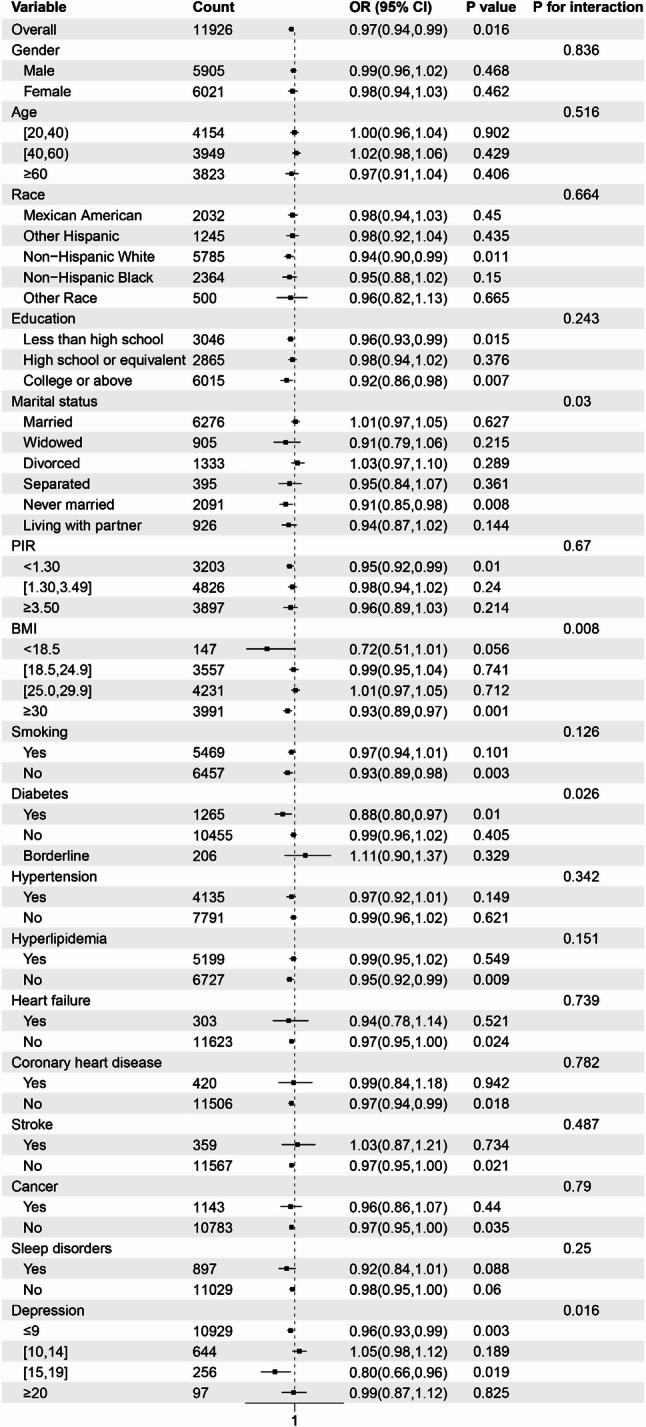
Subgroup analysis demonstrating the relationship between drinking patterns and the risk of diarrhea. The model was adjusted for variables including age, gender, education, marital status, race, PIR, BMI, smoking, hypertension, diabetes, hyperlipidemia, heart failure, coronary heart disease, stroke, cancer, sleep disorders, and depression

The results of the subgroup analysis revealed that participants who were Non-Hispanic White, with lower educational attainment (below high school), never married, from low-income households (PIR < 1.30), with a BMI ≥ 30 kg/m², non-smokers, without diabetes, and free from hyperlipidemia, heart failure, coronary heart disease, stroke, cancer, or depression, exhibited a lower risk of developing diarrhea. Notably, the interaction tests for marital status, BMI, and depression status were statistically significant (*P* < 0.05), indicating that variations in marital status, BMI, and the presence of depression can influence the relationship between drinking patterns and the risk of diarrhea. Detailed data can be found in Fig. 5.

## Discussion

This study systematically examines the relationship between drinking patterns and the incidence of constipation and diarrhea using data from the 2005–2010 NHANES, focusing on the adult population in the United States. Analysis was conducted using multivariate logistic regression. In Model 1, which did not adjust for confounding factors, a negative correlation was observed between the average daily alcohol consumption, treated as a continuous variable, and the risk of constipation. A similar negative correlation was noted with the risk of diarrhea. The drinking level, treated as a categorical variable, exhibits an inverse correlation with the risk of constipation. A similar inverse relationship is observed with the risk of diarrhea. In Models 2 and 3, which adjusted for partial and full confounding factors, the inverse relationship between average daily alcohol consumption and the risk of constipation remained statistically significant. The inverse relationship between drinking level and the risk of constipation was not significant among participants who engaged in light drinking. However, among those who consumed alcohol at moderate to heavy levels, a significant inverse correlation persisted. Furthermore, the results of the trend test demonstrated statistical significance. In contrast, no significant inverse correlation was observed between average daily alcohol consumption and the risk of diarrhea, nor between alcohol consumption levels and diarrhea risk. Notably, in Model 3, light alcohol consumption was associated with a reduced risk of diarrhea, although the trend test showed no statistical significance. The RCS curve analysis revealed a non-linear relationship between average daily alcohol consumption and constipation risk after adjusting for all confounders, indicating that an increase of one drink per day was associated with an elevated risk of constipation. In contrast, no significant non-linear relationship was found between average daily alcohol consumption and the risk of diarrhea. Furthermore, subgroup analyses revealed significant interactions between drinking patterns and the risk of constipation across various demographic and clinical characteristics, including age, race, BMI, and cancer status. Among individuals aged 20–40 years and those aged 60 years and older, the risk of constipation was reduced by 12% and 20%, respectively. Non-Hispanic White participants exhibited an 18% reduction in constipation risk, while participants with a BMI between 18.5 and 24.9 kg/m² experienced a 16% reduction. Additionally, participants without a history of cancer had an 8% lower risk of constipation. Interactions were also observed between drinking patterns and the risk of diarrhea, particularly in relation to marital status, BMI, and depression status. Participants who were never married exhibited a 9% reduction in diarrhea risk, while those with a BMI ≥ 30 kg/m² showed a 7% reduction. Furthermore, individuals without depression demonstrated a 4% lower risk of diarrhea. Our study sheds light on the impact of drinking patterns on gastrointestinal health. Notably, this influence appears to be consistently present among individuals experiencing constipation, largely unaffected by other confounding factors. However, the relationship between drinking patterns and diarrhea risk is more susceptible to the influence of confounders, suggesting the need for further investigation into the specific mechanisms through which drinking patterns affect gut health, as well as the broader implications of these findings for lifestyle choices and human health.

Similarly, Chen et al. found a significant nonlinear negative correlation between daily dietary alcohol intake (g/day) and constipation risk, with the risk of constipation decreasing as daily alcohol consumption increased [16]. Research by Japanese scholars indicates that participants who consumed alcohol more than five days a week, engaged in at least 30 min of exercise twice a week for over a year, and had sufficient sleep exhibited a significantly reduced risk of developing chronic constipation [27]. Several other studies have also suggested that alcohol consumption acts as a protective factor against constipation [28, 29]. The majority of current studies examining the relationship between drinking patterns and the risk of diarrhea indicate that alcohol consumption may either provoke or exacerbate diarrhea. Research by David et al. on the correlation between heavy drinking and traveler’s diarrhea revealed that, during their stay in Mexico, men who consumed more than five drinks per day were at a higher risk of developing diarrhea compared to women with the same level of alcohol intake [30]. Furthermore, studies investigating drinking patterns and gastrointestinal symptoms in patients with IBS have found that, among women with diarrhea-predominant IBS, the association between alcohol consumption and gastrointestinal symptoms is stronger compared to women with constipation-predominant or mixed-type IBS [31]. A study by Wang et al. revealed a significant association between depression and chronic diarrhea, with alcohol consumption further intensifying the correlation between the two conditions [32].

The aforementioned studies indicate a certain relationship between alcohol consumption and intestinal health. The mechanisms underlying this relationship can be explored from several perspectives: (1) Modulation of gut microbiota: Alcohol intake can influence the composition of gut microbiota, alter the intestinal microenvironment, and regulate the digestion and absorption of water and chyme, thereby leading to changes in stool characteristics, which may alleviate either constipation or diarrhea [33–36]. (2) Regulation of intestinal permeability: Alcohol can directly damage intestinal cells and also interfere with tight junction proteins, disrupting their structural integrity. This, in turn, compromises the intestinal barrier, modulating intestinal permeability and impairing water absorption, thus reducing the risk of constipation [37, 38].((3) Regulation of gastrointestinal motility: High concentrations of alcoholic beverages can inhibit gastric motility, whereas low concentrations accelerate gastric emptying. Furthermore, the rapid intake of alcohol in a short period suppresses gastric emptying, while chronic, excessive alcohol consumption enhances gastric motility. The impact of alcohol on small intestinal peristalsis varies depending on the drinking pattern (acute or chronic); acute alcohol consumption impedes small bowel transit, while long-term, heavy drinking accelerates it[39]. These findings indicate that alcohol exerts a bidirectional influence on gastrointestinal motility. (4) Intestinal Inflammation: Alcohol can induce intestinal inflammation through various mechanisms, including altering the composition and function of the gut microbiota, increasing the permeability of the intestinal lining, and disrupting intestinal immune homeostasis. These alterations contribute to an increased frequency of bowel movements and a reduction in stool consistency, thereby lowering the risk of constipation [40]. In summary, the regulation of intestinal health by alcohol may be bidirectional. However, the majority of evidence suggests that alcohol consumption reduces the risk of constipation. The mechanisms by which alcohol may inhibit the occurrence of diarrhea, as well as clinical studies supporting this, remain insufficient and underexplored.

The study presents the following advantages: (1) The data used in this article originate from a U.S. government-affiliated institution, ensuring authoritative and reliable data. The study spans three cycles and includes a large sample size, while accounting for complex sampling analysis, thereby enhancing the credibility of the data.(2) Multivariate logistic regression was employed to explore the relationship between exposure and outcome. By constructing different models and adjusting for relevant confounding factors, the results of the regression analysis are deemed authentic and reliable. Additionally, RCS curves were used to confirm the nonlinear relationship between exposure and outcome.(3) Subgroup analysis was conducted to examine the heterogeneity of effects between alcohol consumption and intestinal health, aiding in the identification of high-risk and specific effect populations. However, the study also has the following limitations: (1) As a cross-sectional study, it cannot directly infer causality, making it difficult to establish the temporal relationship between exposure and outcome.(2) The data were derived from participant questionnaires, which may be subject to recall bias and social desirability bias, potentially compromising data accuracy.(3) The study is limited by the time effect, making it unable to predict long-term trends and changes. To make up for the above shortcomings, future studies can add laboratory data, such as blood alcohol content, to quantify drinking patterns, and repeat measurements to ensure the accuracy of the data.

## Conclusion

In summary, this study analyzed data from the 2005–2010 NHANES to explore the association between drinking patterns (average daily alcohol consumption and drinking intensity) and constipation and diarrhea. The findings suggest a nonlinear negative correlation between alcohol consumption and constipation. This relationship is influenced by factors such as age, race, BMI, and cancer status, and remains stable even after adjusting for various confounding factors. Additionally, a negative correlation was observed between alcohol consumption and diarrhea, though this result is less stable. Although this finding has clinical limitations at the individual level, it enhances our understanding of lifestyle factors in functional gastrointestinal disorders, and future studies should focus on the potential biological mechanisms behind this association and explore whether specific drinking patterns modify this relationship.

## Data Availability

All data in this study are publicly available and traceable, data source: [https://wwwn.cdc.gov/nchs/nhanes/Default.aspx].

## References

[1] Suares NC, Ford AC. Prevalence of, and risk factors for, chronic idiopathic constipation in the community: systematic review and meta-analysis. Am J Gastroenterol. 2011;106(9):1582–91. 10.1038/ajg.2011.164. 21606976 10.1038/ajg.2011.164

[2] Barberio B, Judge C, Savarino EV, Ford AC. Global prevalence of functional constipation according to the Rome criteria: a systematic review and meta-analysis. Lancet Gastroenterol Hepatol. 2021;6(8):638–48. 10.1016/S2468-1253(21)00111-4. 34090581 10.1016/S2468-1253(21)00111-4

[3] Scott SM, Simrén M, Farmer AD, et al. Chronic constipation in adults: contemporary perspectives and clinical challenges. 1: Epidemiology, diagnosis, clinical associations, pathophysiology and investigation. Neurogastroenterol Motil. 2021;33(6):e14050. 10.1111/nmo.14050. 33263938 10.1111/nmo.14050

[4] Aziz I, Whitehead WE, Palsson OS, Törnblom H, Simrén M. An approach to the diagnosis and management of Rome IV functional disorders of chronic constipation. Expert Rev Gastroenterol Hepatol. 2020;14(1):39–46. 10.1080/17474124.2020.1708718. 31893959 10.1080/17474124.2020.1708718

[5] Palsson OS, Whitehead WE, van Tilburg MAL, et al. Development and validation of the Rome IV diagnostic questionnaire for adults. Gastroenterology. 2016;150(6):1481–91. 10.1053/j.gastro.2016.02.014. 10.1053/j.gastro.2016.02.014 27144634

[6] Bharucha AE, Lacy BE. Mechanisms, Evaluation, and management of chronic constipation. Gastroenterology. 2020;158(5):1232–e12493. 10.1053/j.gastro.2019.12.034. 31945360 10.1053/j.gastro.2019.12.034 PMC7573977

[7] Nag A, Martin SA, Mladsi D, Olayinka-Amao O, Purser M, Vekaria RM. The humanistic and economic burden of chronic idiopathic constipation in the USA: a systematic literature review. Clin Exp Gastroenterol. 2020;13:255–65. 10.2147/CEG.S239205. 32765039 10.2147/CEG.S239205 PMC7371558

[8] Sanchez MIP, Bercik P. Epidemiology and burden of chronic constipation. Can J Gastroenterol Hepatol. 2000;25:B11–5. 10.1155/2011/125491. 10.1155/2011/974573 PMC3206560 22114752

[9] Kyu HH, Vongpradith A, Dominguez RMV et al. Global, regional, and national age-sex-specific burden of diarrhoeal diseases, their risk factors, and aetiologies, 1990–2021, for 204 countries and territories: a systematic analysis for the Global Burden of Disease Study 2021. The Lancet Infectious Diseases. 2024;0(0). 10.1016/S1473-3099(24)00691-1. 10.1016/S1473-3099(24)00691-1 PMC12018300 39708822

[10] Arasaradnam RP, Brown S, Forbes A, et al. Guidelines for the investigation of chronic diarrhoea in adults: British Society of Gastroenterology, 3rd edition. Gut. 2018;67(8):1380–99. 10.1136/gutjnl-2017-315909. 29653941 10.1136/gutjnl-2017-315909 PMC6204957

[11] Sandler RS, Stewart WF, Liberman JN, Ricci JA, Zorich NL. Abdominal pain, bloating, and diarrheain the United States. Dig Dis Sci. 2000;45(6):1166–71. 10.1023/A:1005554103531. 10877233 10.1023/a:1005554103531

[12] Zhao YF, Guo XJ, Zhang ZS, et al. Epidemiology of functional diarrhea and comparison with diarrhea-Predominant irritable bowel syndrome: A Population-Based survey in China. PLoS ONE. 2012;7(8):e43749. 10.1371/journal.pone.0043749. 22937091 10.1371/journal.pone.0043749 PMC3427143

[13] Global. regional, and National age-sex specific mortality for 264 causes of death, 1980–2016: a systematic analysis for the global burden of disease study 2016. Lancet. 2017;390(10100):1151–210. 10.1016/S0140-6736(17)32152-9. 28919116 10.1016/S0140-6736(17)32152-9 PMC5605883

[14] Ritchie H, Roser M. Alcohol Consumption. Our World in Data. Published online January 1, 2022. Accessed April 1, 2025. https://ourworldindata

[15] Goldman MR, Molina-Castro M, Etkins JC, et al. Recent advances in alcohol metabolism: from the gut to the brain. Physiol Rev. 2025;105(4):2501–35. 10.1152/physrev.00053.2024. 40637545 10.1152/physrev.00053.2024 PMC12345593

[16] Chen WX, Peng XF, Yu M, Wang DC. Daily alcohol intake and its negative association with constipation based on NHANES data 2005–2010. Sci Rep. 2025;15(1):10021. 10.1038/s41598-025-91899-9. 40122926 10.1038/s41598-025-91899-9 PMC11930976

[17] Shiotani A, Ishikawa H, Mutoh M, et al. Impact of diarrhea after drinking on colorectal tumor risk: a case control study. Asian Pac J Cancer Prev. 2019;20(3):795–9. 10.31557/APJCP.2019.20.3.795. 30909690 10.31557/APJCP.2019.20.3.795 PMC6825756

[18] Kong W, Sheng W, Zheng Y. Modification of the association between coffee consumption and constipation by alcohol drinking: a cross-sectional analysis of NHANES 2007–2010. PLoS One. 2024;19(10):e0311916. 10.1371/journal.pone.0311916. 39453914 10.1371/journal.pone.0311916 PMC11508157

[19] Engen PA, Green SJ, Voigt RM, Forsyth CB, Keshavarzian A. The gastrointestinal microbiome. Alcohol Res. 2015;37(2):223–36. 26695747 10.35946/arcr.v37.2.07 PMC4590619

[20] Questionnaires NHANES, Datasets, and, Documentation R. Accessed April 2, 2025. https://wwwn

[21] Probert CJ, Emmett PM, Heaton KW. Intestinal transit time in the population calculated from self made observations of defecation. J Epidemiol Community Health. 1993;47(4):331–3. 8228773 10.1136/jech.47.4.331 PMC1059804

[22] Yang X. Association between drinking patterns and diabetic kidney disease in United States adults: a cross-sectional study based on data from NHANES 1999–2016. Ren Fail. 2025;47(1):2454970. 10.1080/0886022X.2025.2454970. 39842843 10.1080/0886022X.2025.2454970 PMC11755733

[23] Jiang M, Tang X, Wang P, Yang L, Du R. Association between daily alcohol consumption and serum alpha Klotho levels among U.S. adults over 40 years old: a cross-sectional study. BMC Public Health. 2023;23:1901. 10.1186/s12889-023-16830-1. 37784055 10.1186/s12889-023-16830-1 PMC10544600

[24] U.S. Department of Health & Human Services. Poverty Guidelines, Research, and Measurement. 2011. http://aspe

[25] Kroenke K, Spitzer RL, Williams JB. The PHQ-9: validity of a brief depression severity measure. J Gen Intern Med. 2001;16(9):606–13. 10.1046/j.1525-1497.2001.016009606.x. 11556941 10.1046/j.1525-1497.2001.016009606.x PMC1495268

[26] Body mass index (BMI). Accessed August 31. 2025. https://www

[27] Otani K, Watanabe T, Takahashi K, et al. Prevalence and risk factors of functional constipation in the Rome IV criteria during a medical check-up in Japan. J Gastroenterol Hepatol. 2021;36(8):2157–64. 10.1111/jgh.15436. 33555082 10.1111/jgh.15436

[28] Yang C, Hong Q, Wu T, Fan Y, Shen X, Dong X. Association between dietary intake of live microbes and chronic constipation in adults. J Nutr. 2024;154(2):526–34. 10.1016/j.tjnut.2023.11.032. 38072155 10.1016/j.tjnut.2023.11.032

[29] Huang X, Zhao L, Li Z, Gu X, Li M, Xiang J. Association of niacin intake with constipation in adult: result from the National Health and Nutrition Examination. Eur J Med Res. 2023;28(1):377. 10.1186/s40001-023-01362-6. 37752534 10.1186/s40001-023-01362-6 PMC10523733

[30] Huang DB, Sanchez AP, Triana E, Jiang ZD, DuPont HL, Ericsson CD. United States male students who heavily consume alcohol in Mexico are at greater risk of travelers’ diarrhea than their female counterparts. J Travel Med. 2006. 10.2310/7060.2004.18560. 10.2310/7060.2004.18560 15710056

[31] Reding KW, Cain KC, Jarrett ME, Eugenio MD, Heitkemper MM. Relationship Between Patterns of Alcohol Consumption and… Official journal of the American College of Gastroenterology| ACG. Accessed April 9, 2025. https://journals.lww.com/ajg/abstract/2013/02000/relationship_between_patterns_of_alcohol.23.aspx.

[32] Wang Y, Li X, Cao Z, Zhou Y. The impact of alcohol consumption on the relationship between depression and chronic diarrhea: a cross-sectional study analysis on NHANES (2005–2010). Front Psychiatry. 2024;15:1393546. 10.3389/fpsyt.2024.1393546. 39279809 10.3389/fpsyt.2024.1393546 PMC11392863

[33] Engen PA, Green SJ, Voigt RM, Forsyth CB, Keshavarzian A. The gastrointestinal microbiome: alcohol effects on the composition of intestinal microbiota. Alcohol Res. 2015;37(2):223–36. 26695747 10.35946/arcr.v37.2.07 PMC4590619

[34] Qamar N, Castano D, Patt C, Chu T, Cottrell J, Chang SL. Meta-analysis of alcohol induced gut dysbiosis and the resulting behavioral impact. Behav Brain Res. 2019;376:112196. 10.1016/j.bbr.2019.112196. 31476330 10.1016/j.bbr.2019.112196

[35] Liu L, Nguyen SM, Wang L, et al. Associations of alcohol intake with gut microbiome: a prospective study in a predominantly low-income Black/African American population. Am J Clin Nutr. 2025;121(1):134–40. 10.1016/j.ajcnut.2024.11.007. 39537028 10.1016/j.ajcnut.2024.11.007 PMC11747185

[36] Caslin B, Mohler K, Thiagarajan S, Melamed E. Alcohol as friend or foe in autoimmune diseases: a role for gut microbiome? Gut Microbes. 2021;13(1):1916278. 10.1080/19490976.2021.1916278. 34224314 10.1080/19490976.2021.1916278 PMC8259720

[37] Khoshbin K, Camilleri M. Effects of dietary components on intestinal permeability in health and disease. American journal of physiology-Gastrointestinal and liver physiology. 2020. 10.1152/ajpgi.00245.2020. 32902315 10.1152/ajpgi.00245.2020 PMC8087346

[38] Diao XY, Peng T, Kong FG, et al. Alcohol consumption promotes colorectal cancer by altering intestinal permeability. Eur Rev Med Pharmacol Sci. 2020;24(18):9370–7. 10.26355/eurrev_202009_23020. 33015778 10.26355/eurrev_202009_23020

[39] Grad S, Abenavoli L, Dumitrascu DL. The effect of alcohol on gastrointestinal motility. Rev Recent Clin Trials. 2016;11(3):191–5. 10.2174/1574887111666160815103251. 27527893 10.2174/1574887111666160815103251

[40] Bishehsari F, Magno E, Swanson G, et al. Alcohol and Gut-Derived inflammation. Alcohol Res. 2017;38(2):163–71. 28988571 10.35946/arcr.v38.2.02 PMC5513683

